# Resynthesizing behavior through phylogenetic refinement

**DOI:** 10.3758/s13414-019-01760-1

**Published:** 2019-06-03

**Authors:** Paul Cisek

**Affiliations:** Department of Neuroscience, University of Montréal, Montréal, Québec Canada

**Keywords:** Cognitive neuroscience, Animal cognition, Neural mechanisms, Evolution

## Abstract

This article proposes that biologically plausible theories of behavior can be constructed by following a method of “phylogenetic refinement,” whereby they are progressively elaborated from simple to complex according to phylogenetic data on the sequence of changes that occurred over the course of evolution. It is argued that sufficient data exist to make this approach possible, and that the result can more effectively delineate the true biological categories of neurophysiological mechanisms than do approaches based on definitions of putative functions inherited from psychological traditions. As an example, the approach is used to sketch a theoretical framework of how basic feedback control of interaction with the world was elaborated during vertebrate evolution, to give rise to the functional architecture of the mammalian brain. The results provide a conceptual taxonomy of mechanisms that naturally map to neurophysiological and neuroanatomical data and that offer a context for defining putative functions that, it is argued, are better grounded in biology than are some of the traditional concepts of cognitive science.

A major challenge of any scientific endeavor is not only to provide good answers to the questions we ask about our world, but to find good questions to ask in the first place. This is difficult, because defining the questions must perforce be done at the start of a research program, before we can really be sure we have formulated them properly. In general, we inherit the questions of our intellectual predecessors, who knew even less than we do, and thus risk seeking explanations for concepts that were not defined in a manner that best captures the real processes of interest. Consequently, we are motivated to periodically reexamine the questions we aim to answer and to look outside whatever field we have defined ourselves into.

Although there is no single set of concepts that all brain scientists agree upon, it is possible to summarize current mainstream thinking in terms of a conceptual taxonomy like that shown in Fig. 1. According to this view, behavior is composed of perceptual, cognitive, and action systems, each of which can be further decomposed (Hurley, 2001) into subsystems and subfunctions, such as object recognition, attention, episodic memory, economic decision-making, forward models, and so forth. These are the questions that define research programs—the explananda that one seeks to explain, ultimately at a neurophysiological level. Consequently, although few would claim that these functions are strictly independent, they tend to be studied by separate independent investigators, often leading to their reification as distinct entities whose biological substrate is assumed to be identifiable (Hommel & Colzato, 2015).

**Fig. 1:**
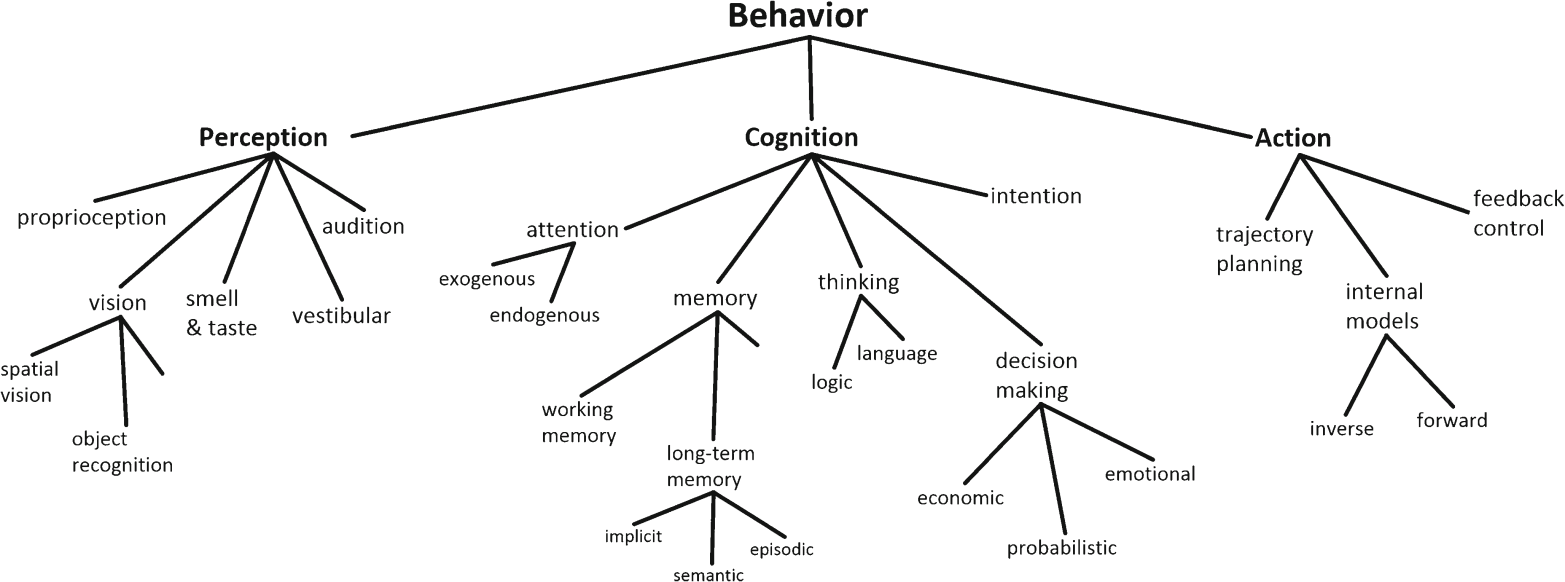
Partial sketch of a conceptual taxonomy implicit in mainstream cognitive science and neuroscience

However, the task of mapping the concepts of Fig. 1 to neural data has proven difficult. The neural correlates of putatively unified functions, such as “working memory” or “decision making,” appear to be distributed throughout the brain, whereas other concepts that one might expect to be separate, such as “attention” and “intention,” are often intermixed in single regions, sometimes even at the level of single cells (Cisek & Kalaska, 2010). The bridge between psychological concepts and neurophysiological mechanisms is difficult to establish, leading to proposals that many of the questions being asked are perhaps not ideally framed (Cisek & Kalaska, 2010; Hommel et al., 2019; Hommel & Colzato, 2015; Hurley, 2001; Lebedev & Wise, 2002; Lindquist & Barrett, 2012).

For example, let’s consider one fundamental concept defining many research programs in cognitive neuroscience—that of “cognition” itself. In the weakest sense, this is just a noun used to distinguish processes that make use of explicit representations of knowledge versus those that do not. Often, however, the term is used in a stronger sense, implying a system in the brain that performs a specific set of functions. According to mainstream views, this system is separate from perception and action (Firestone & Scholl, 2016; Fodor, 1983; Pylyshyn, 1984) and lies interposed between the two (Hurley, 2001), governing what we colloquially consider mental processes. But where does that concept come from? Whom do we cite when we propose cognition as the starting point for defining our research projects? Many accounts emphasize a “cognitive revolution” (G. A. Miller, 2003) that began in the 1950s with the Dartmouth conference (McCarthy, Minsky, Rochester, & Shannon, 1955), or Chomsky’s (1959) critique of Skinner, but the fundamental ideas about representation and computation date back at least to Thomas Hobbes (1588–1679), George Berkeley (1685–1753), and John Stuart Mill (1773–1836). Furthermore, the functional architecture whereby cognition sits between perception and action (Fig. 1) is much older still. The concepts of distinct perception and action systems were forced upon early philosophers such as Plato (425–347 BCE) and Descartes (1596–1650) by their belief in a nonphysical mind: If the mind is nonphysical, then interfaces must exist between it and the physical world. Perception is what presents the world to the mind, and action is what plays out the mind’s (free) will upon the world. Although the concept of the nonphysical mind has been replaced by a physical computational process of cognition, the conceptual architecture has remained (Cisek, 1999). But if we’ve abandoned dualism, is it also time to abandon that serial architecture, as well as the concept of a cognitive system at its center? What would be the alternative?

That last question above is a challenging one: What would be the alternative to cognition? Any student of psychological history knows that many alternatives have been proposed for many years, with “embodied cognition” recently rising into prominence (Clark, 1997; Engel, Maye, Kurthen, & Konig, 2013; Klatzky, Behrmann, & MacWhinney, 2008; Pezzulo & Cisek, 2016; Wilson, 2002). The key debates often focus on the issue of representations: Do they exist? What form do they take? How much of behavior can or cannot be explained if we just do away with them entirely? At one extreme, it has been argued that representations do not exist in the brain at all (Brette, 2017; Chemero, 2009; Gibson, 1979; O’Regan & Noe, 2001). But some say this cannot be the case, since representations (even symbolic ones) are clearly used in human language—such as when writing articles about embodied cognition.

The arguments for and against “cognition” and “representation” are difficult to disentangle and will probably continue for many years. It is likely that both camps will turn out to be partially correct, and that the truth will be seen to lie somewhere between or beside them. In the meantime, however, a different approach can be taken to yield insights into the biological organization of behavior. That approach is to take advantage of something we can all agree on, and what is possibly the most important thing we know about the brain: that it evolved.

## Resynthesis through phylogenetic refinement

The study of evolution is a complex field of research, unfamiliar to many in the cognitive sciences. That is understandable, since the task of figuring out how the brain works is already a monumental challenge. Francis Crick (1994) himself once said that “any reasonable way to go about finding out how a mechanism evolved would be first to find out how the mechanism works, and then worry about how it evolved.” However, I respectfully disagree. Not only does evolution provide just the guidance we need for inferring the biological organization of behavior, but there is little hope of doing so without it. As Dobzhansky (1973, p. 125) famously said, “Nothing in biology makes sense except in the light of evolution.” Consequently, figuring out how the brain works might be much easier if we know something about how it evolved.

One reason why understanding evolution is important is that the manner in which it constructs biological systems is very different from what human engineers do when building artificial systems for performing useful functions. In engineering, one begins by precisely defining a challenge to be solved, and then devising and testing plausible mechanisms for solving it. In contrast, evolution does not identify challenges at all. Instead, it modifies the developmental process of individuals within populations and then, through natural selection, favors those variations that happen to accomplish something that *used to be* a challenge. The sequence of changes accumulated over millions of years is never directed in any way, never aimed at any functional purpose, even though it may ultimately achieve amazing things. Consequently, the organization of the resulting systems is not determined as much by categories of functional challenges as by the constraints of the kinds of developmental changes that are possible at each stage of evolution. This means that the majority of sensible mechanisms that may be optimal in some way never even entered into the game, rendering normative analysis ineffective. Furthermore, even *defining* what the functional challenges are (e.g., Fig. 1) is fraught with pitfalls, because each subsystem we may find in modern animals is always a specialization that emerged within a broader ancestral system, and even modern humans are a “work in progress.” That is why evolutionary history is important. As Hendriks-Jansen (1996, p. 8) put it, “functional decomposition and natural selection do not mix. . . . The only appropriate explanation for a piece of behavior resulting from natural selection is an explanation in terms of its historical emergence within a succession of species-typical environments.”

Although the discussion above may seem pessimistic, and seem to imply that we have little hope of ever making sense of the brain, I believe just the opposite is true. Because evolution is so constrained in the kinds of changes it can introduce into a population of animals, the resulting sequence of modifications is highly conservative. Each evolutionary innovation, no matter how advantageous it may prove to be, must first be *possible* within the context of the ancestral system. Furthermore, it must be a change in the developmental process that does not interfere with later stages of development. For this reason, early stages of ontogeny are often very similar between species—because mutations in these stages are likely to violate assumptions upon which the rest of development works. This dramatically limits the kinds of modifications that can enter into natural selection and also limits how far two species can diverge away from their common ancestor. Such constraints make comparative biology possible, whereby insights into evolution can be inferred from comparing species whose phylogenetic relationships are known. Thus, although it may not be possible for us to know exactly *why* a given lineage changed in the way it did, we can infer *how* it changed. That is, we can reconstruct the sequence of innovations along a given lineage in enough detail to constrain our theories of biological mechanisms in a manner that reflects the actual process that generated those mechanisms. What should be encouraging to those of us trying to understand the brain is that a great deal of data already exist to make this approach possible.

The central goal of this article is to demonstrate an approach to building psychological theories, which we can call “phylogenetic refinement.” The idea is to progressively refine theories of behavior, whereby each hypothetical mechanism is conceived as an extension of an ancestral one, and whenever possible, each extension is guided by data on the actual phylogenetic history. The emphasis is always on processes rather than systems, and neuroanatomical continuity is taken as a guiding principle. This progressive approach is even used to define the questions we ask and to delineate distinctions between different abilities or neural subsystems. For example, instead of defining cognition as the set of abilities that are separate from sensorimotor control (Fodor, 1983; Pylyshyn, 1984), we can instead ask how a meaningful distinction between cognitive and sensorimotor processes could have emerged, at an empirically definable time in history, within a unified ancestral system for governing adaptive behavior. In a biological organism, any distinction between two subsystems has to be compatible with a history of how they differentiated from something that was unified, and this history will naturally lead us to develop conceptual taxonomies that reflect true biological categories. As I emphasize below, there is already a remarkable amount of knowledge about the sequence of changes along the evolutionary lineage that led to humans, and despite various remaining debates, a basic picture is emerging that can be a powerful guide for building theories of how our brain implements behavior.

Although most research in cognitive science and neuroscience tends not to take advantage of evolutionary data, the idea of using such data to guide theory development is not by any means new. It lies at the core of both ethology (Hinde, 1966) and comparative biology (Butler & Hodos, 2005); it is the basic philosophy of behavior-based robotics (Brooks, 1991; Hendriks-Jansen, 1996) and their application to interpreting the brain (Prescott, Redgrave, & Gurney, 1999); and it underlies the computational models of Grossberg and colleagues (e.g., Grossberg, 1978). Many authors have made extensive use of evolutionary considerations to construct theories of memory (Murray, Wise, and Graham, 2017), executive function (Passingham & Wise, 2012), and even consciousness (Feinberg & Mallatt, 2016; Lacalli, 2018a; Merker, 2005). One can take this attitude still further and suggest that not only can we use phylogenetic history to help us find the answers to the questions we ask about behavior, but we can use it to define what the right questions are in the first place (Hendriks-Jansen, 1996). In other words, we can use it to resynthesize the conceptual taxonomy that delineates the “natural kinds” we seek to explain. Ideally, that new taxonomy will progressively differentiate functions and subfunctions in a way that mirrors their progressive differentiation over evolutionary time, and the hope is that the resulting set of explananda will more naturally correspond to real biological circuits.

**Fig. 2:**
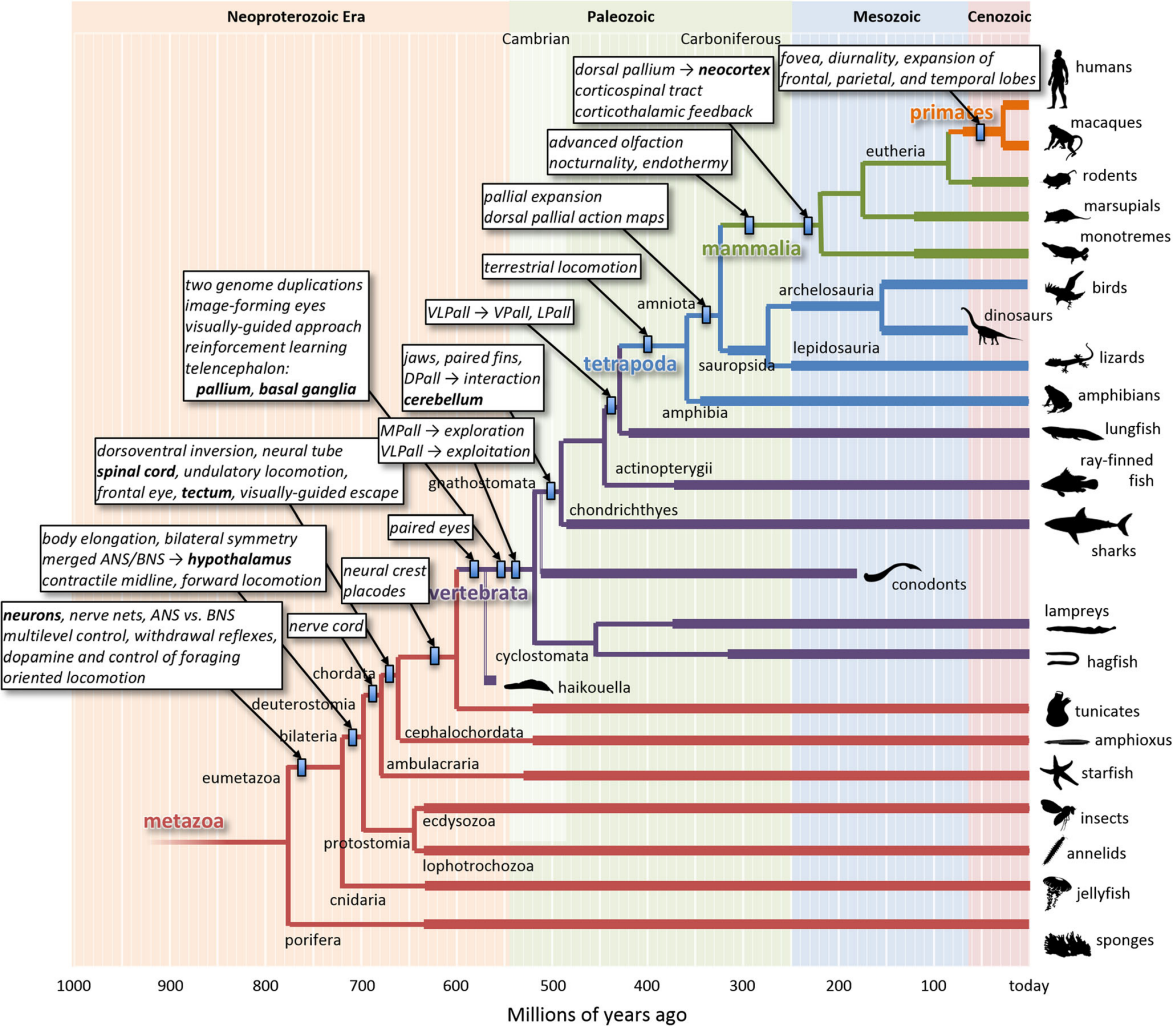
Phylogenetic tree of animals, emphasizing the lineage that led to humans. Branch points represent some of the divergences between different lineages, with timing estimated using molecular-clock analyses (Erwin et al., 2011; Wray, 2015). Thick lines indicate the presence of relevant fossil data (https://paleobiodb.org). Small rectangles indicate the latest estimated timing of the innovations described in the boxes. Many branch points and lineages are omitted for clarity. The silhouettes of example species are from http://phylopic.org. Note that the arrangement of the branches in the vertical direction is completely arbitrary. Here it is arranged so as to leave room to emphasize branch points and innovations along the lineage leading to humans, and this is the only reason for the apparent “Scala Naturae” of species along the right edge. ANS, apical nervous system; BNS, blastoporal nervous system; DPall, dorsal pallium; MPall, medial pallium; LPall, lateral pallium; VLPall, ventrolateral pallium; VPall, ventral pallium

Here I use phylogenetic refinement to synthesize a taxonomy of the neural mechanisms that govern simple sensorimotor control in vertebrates, setting out the ancestral context that can serve as the baseline set of constraints for more detailed theories of behavior. This is a large goal, and the present article will merely present a basic sketch. Some details of the sketch may turn out to be incorrect, but many are already so well-constrained by a vast collection of behavioral, physiological, anatomical, and developmental data that a first pass seems worthwhile to attempt. Figure 2 presents a “roadmap” that will guide the rest of this article.

## Early behavioral systems

Current theories posit that life on Earth began approximately 3.6 billion years ago in an “RNA world,” with the appearance of chemical reactions that created closed loops called *autocatalytic sets* (Copley, Smith, & Morowitz, 2007; Hordijk, Hein, & Steel, 2010; Joyce, 2002; Kauffman, 1993; Vaidya et al., 2012). These simple chemical systems possessed two key properties: They maintained their own structure by continuously resynthesizing their own elements (metabolism), and they produced copies of themselves in the immediate vicinity (replication). These properties were sufficient to launch evolution, leading to additional innovations such as the emergence of DNA (Bedian, 1982; Crick, Brenner, Klug, & Pieczenik, 1976) and the enclosing of metabolic loops within membranes (Fox, 1965), culminating in the appearance of cells. I will not describe these important events in any detail, except to note that from the very beginning, one key function of living systems was metabolism, which was accomplished through “closed-loop” biochemical control that maintained the organism within a range of desirable states—for example, a chemically rich internal environment, an intact membrane, and so forth (Maturana & Varela, 1980). It is possible for evolution to establish this type of control because it takes advantage of the reliable laws of physics and chemistry, and today we call such internal control mechanisms “physiology.”

The control loops that keep the organism within a range of desirable states need not be entirely contained within the membrane. For example, some types of chemicals cannot be synthesized internally, but must be absorbed from the external environment. However, these nutrients are not uniformly distributed in the world. Hence, if an organism finds itself in a nutrient-poor local environment, it may improve its situation simply by moving randomly (perhaps waving cilia or a flagellum), and this is likely to bring it into a richer environment where more of the nutrient can be absorbed, thereby improving the internal state. Importantly, it is also possible for evolution to establish this type of control because it takes advantage of reliable laws of the statistics of nutrient distributions. Such control mechanisms, which extend into the environment, can be called “behavior” (Ashby, 1965; Cisek, 1999; Maturana & Varela, 1980; Powers, 1973).

From this perspective, it is not surprising that neurons first appeared at the interface between the body and the world as a specialization of the external cell layer of multicellular animals (Brunet & Arendt, 2016; Jekely, 2011; Mackie, 1970). This occurred approximately 750–800 million years ago (Mya), after our lineage split from that leading to sponges (Ereskovsky & Dondua, 2006). Initially these cells were “pluripotent” and played both sensory and motor roles. For example, some responded to mechanical deformation at their distal end with a mechanical contraction at their proximal end, thereby causing the body to withdraw from undesirable contact. Others contained chemical and photosensitive receptors and produced changes in the rest of the body through the secretion of hormones. Over time, different cells specialized to emphasize the sensory and motor roles, while others became specialized at coordinating signals in a network across the body, and today we call these “neurons.”

**Fig. 3:**
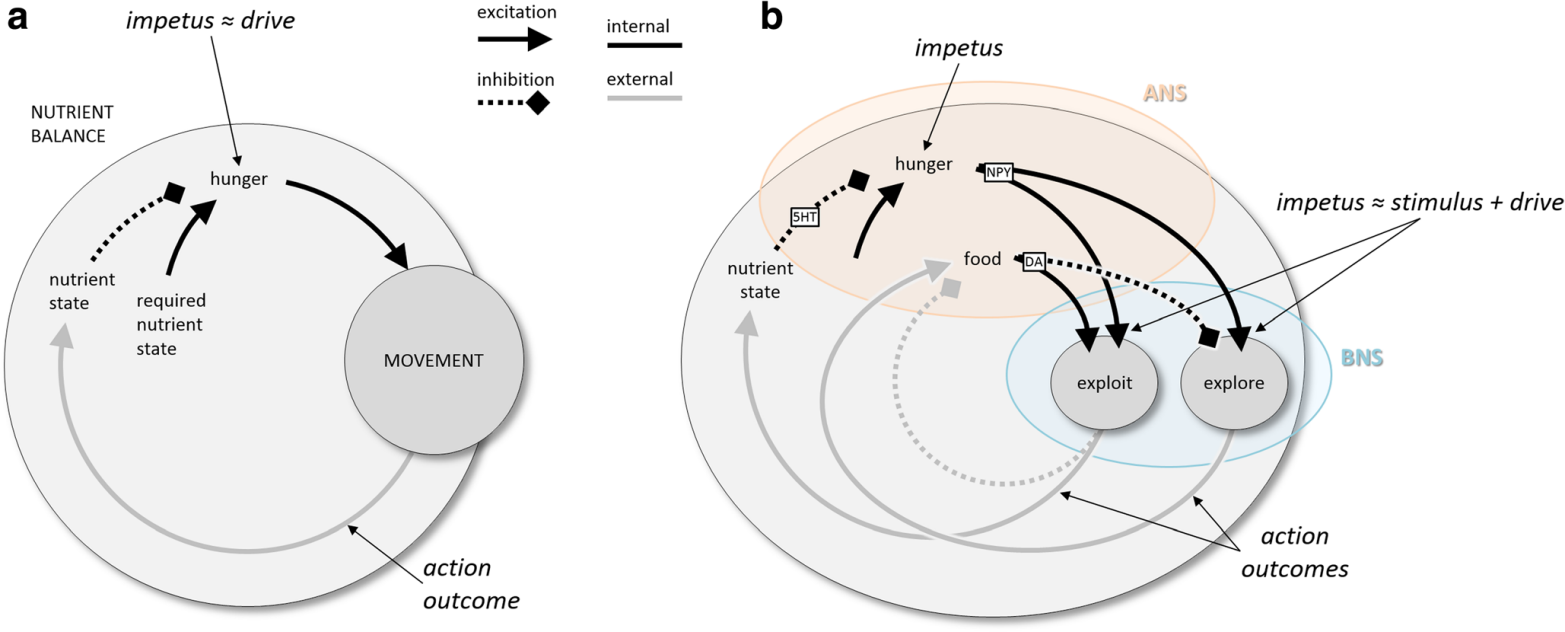
Schematic behavioral control systems. (A) When the current nutrient state deviates from a desired state, locomotion is initiated, ultimately bringing the animal to a more desirable state. (B) Elaboration of nutrient state control into a high-level controller (ANS) and a lower-level controller (BNS) capable of two modes of locomotion, local exploitation, and long-range exploration. 5HT, serotonin; ANS/BNS, apical/blastoporal nervous system; DA, dopamine; NPY, neuropeptide Y

Figure 3A presents a simple schematic of a basic behavioral control system, along with some definitions, using nutrient control as an example. At its heart is an evaluation of the animal’s current state in relation to a range of desirable states. Deviations of the nutrient state outside the desirable range constitute the motivation for actions that improve the state. In the case of nutrient control in the earliest multicellular animals (“eumetazoans”), the action was simple random locomotion. Under the assumption of nonuniform nutrient distributions, this tended to result in improving the nutrient state and bringing it back into the desirable range. Note that the result is a *negative* feedback system: The action is performed to eliminate the conditions that motivated the action. Let us define a concept called “impetus,” which refers to the *conditions that motivate actions*, whereby the actions tend to result in reducing or eliminating the impetus. In the given example, the impetus is internal to the animal—a deviation of the nutrient state from a desirable range. We could call it a “hunger drive” (Hull, 1943), and it motivates actions that reduce hunger by improving the nutrient state. In other examples, the impetus may be external. For example, the proximity of a threat is an impetus that motivates escape actions, which reduce the impetus by moving the agent away from the threat. Here the impetus is related to the concept of a “stimulus.” In other words, the idea of the impetus is a generalization of the concepts of internal drives, incentive motivation, as well as sensations of the external world. It does not necessarily imply a representation, or an explicit comparison operation, or even internal variables that correlate with the external world. It merely implies that actions are taken to reduce the conditions that motivate those actions—that is, that behavior functions as a negative feedback loop. This is trivial for describing food-seeking, but it is also useful for describing more complex biological control, including communication (Cisek, 1999). One of the key proposals of the present article is that *the evolutionary history of the nervous system is essentially a history of the continuous extension of such control further and further into the world*.

The early eumetazoans were shaped like inverted cups resembling the blastula phase of development, with an apical end at the top and a blastopore at the bottom, which permitted water inflow and outflow. Neurons distributed across this body specialized into two separate systems (Arendt, Tosches, & Marlow, 2016; Tosches & Arendt, 2013). At the top was an “apical nervous system” (ANS), which was rich in chemo- and photosensitive cells and controlled the basic behavioral state (e.g., energy homeostasis, sleep vs. wake) through hormonal secretions. At the other end, surrounding the blastopore with a ring of sensory and contractile cells was a “blastoporal nervous system” (BNS), which controlled oscillatory contractions that produced either water intake or propulsion through rapid outflow.

Here it becomes useful to make a distinction between two hierarchical levels within the nutrient control system (Fig. 3B), which probably emerged more than 750 Mya. The ANS possessed chemical receptors sensitive to the nutrient content of the external world, which secreted hormones when that content was high. This defined a new lower-level impetus, “hunger + presence of food,” which motivated patterns of oscillatory activity at the blastopore that resulted in water intake. The result implemented filter-feeding, a type of “local exploitation,” which had two consequences. First, it depleted the food supply, reducing its own impetus. Second, it improved the internal nutrient state, reducing the higher-level impetus of “hunger.” If the food was depleted before the hunger impetus was eliminated, the animal would now be in a state of “hunger + no food,” which would motivate oscillatory patterns at the blastopore that resulted in propulsion and, thus, long-range locomotion. This is analogous to “long-range exploration” and tended to have the effect of bringing the animal to sites of higher nutrient concentration. This again led to the exploit action, and the process would continue until the nutrient state was brought into the desirable range, and thus the higher-level impetus of “hunger” was eliminated.

Although the scenario described above is hypothetical, widespread evidence supports many of its features, including the signals involved in the internal causal pathways. In early eumetazoans, the high-level state was governed by the ANS, which in vertebrates became the hypothalamus, whereas specific behavioral policies (such as locomotion) were controlled by the BNS, which became the rest of the nervous system. Even in modern animals, much of the high-level control is still regulated by the hypothalamus. The general state of satiation is signaled by serotonin (Azmitia, 2007, 2010; Voigt & Fink, 2015), and when this falls below a desirable range, the signal for hunger involves neuropeptide Y (Billington & Levine, 1992; Meister, 2007; Minor, Chang, & de Cabo, 2009; Schwartz, Woods, Porte, Seeley, & Baskin, 2000; Voigt & Fink, 2015), a highly conserved transmitter. The shift between local exploitation and long-range exploration is governed by the neurotransmitter dopamine, whose secretion is related to food intake and which stimulates behaviors leading to “area-restricted search” in very diverse species, including nematodes, flatworms, mollusks, and vertebrates (Barron, Sovik, & Cornish, 2010; Hills, 2006). Indeed, as was proposed by Hills et al. (2015), the original role of dopamine may have been the arbitration of motor behavior between local exploitation and long-range exploration, and this was only later elaborated into the neurotransmitter’s many other roles in the vertebrate nervous system, such as the signaling of positive reinforcement (Schultz, 2004) and the modulation of novelty seeking (Costa, Tran, Turchi, & Averbeck, 2014). The resulting pattern of locomotion described above, in which short bursts of local exploitation are interspersed with bouts of long-range exploration, is known as a *Lévy walk* (Sims et al., 2014). Despite its simplicity, it is a highly efficient means of foraging, and it is observed in the behavior of microorganisms, insects, mollusks, reptiles, fish, birds, and even human hunter–gatherers (Humphries et al., 2010; Reynolds, 2015).

Note that every part of the system depicted in Fig. 3B is a feedback loop, each composed of processes within the organism (black lines) as well as actions on the environment around the organism (gray lines). In each case, every complete loop is self-limiting (i.e., the total sign around each circuit is negative), always ultimately reducing its own impetus. Even the double loop that alternates between bouts of exploiting and exploring is nested within a negative feedback loop that ultimately reduces the impetus of hunger that drives the entire behavior. It is this organization into negative feedback loops that makes the behaviors adaptive and gives purpose to their execution. Ignoring that circular nature of control—that is, looking only at the black lines on the diagram—would lead to an impoverished “stimulus–response” view of behavior that fails to capture its biological purpose and adaptive organization. This was exactly the point made by John Dewey over 120 years ago: “What we have is a circuit, not an arc or broken segment of a circle. This circuit is more truly termed organic than reflex, because the motor response determines the stimulus, just as truly as sensory stimulus determines movement” (Dewey, 1896, p. 363).

## Elaboration of behavior along the vertebrate lineage

**Fig. 4:**
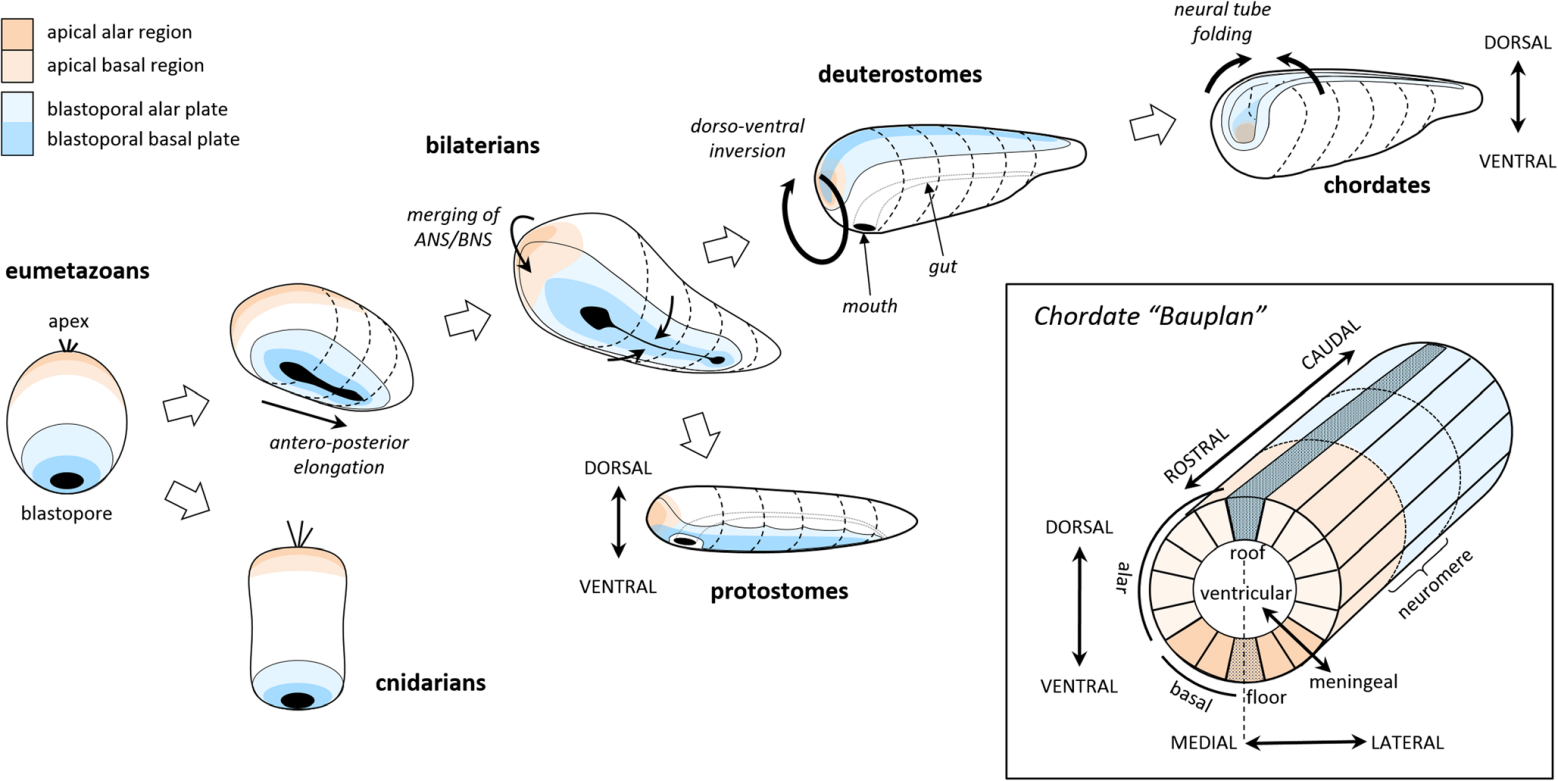
Sequence of changes in early nervous systems leading to the basic plan of chordates. Evolutionary time along the lineage leading to vertebrates is indicated from left to right, with cnidarians (jellyfish, anemones, etc.) and protostomes (annelids, insects, mollusks, etc.) diverging along the way. The inset shows the basic organization of the chordate nervous system and its topological axes, based on Nieuwenhuys and Puelles (2016)

Figure 4 illustrates how the eumetazoan nerve nets evolved into the neural tube between 750 and 650 Mya. The legacy of the distinction between the ANS and BNS can still be found in cnidarians, such as jellyfish and anemones. In our lineage, the bilaterians, the body became elongated, stretching the blastopore into a slit, which then fused in the center to form a digestive tract. At one end of the body, the ANS and BNS merged (Arendt et al., 2016; Tosches & Arendt, 2013) into what would eventually become the head and a centralized brain—although only in a few lineages, such as arthropods, annelids, some mollusks, and chordates (Northcutt, 2012). The rest of the BNS remained on the ventral side of the body in protostomes (annelids, mollusks, arthropods, etc.), but in our branch, the deuterostomes, the body inverted such that the entire nervous system now became dorsally oriented and separate from the mouth and digestive tract (Holland, 2015; Lowe et al., 2006). Finally, in chordates, the nervous system folded inward into the body, forming what defines the basic neural plan to the present day (Nieuwenhuys & Puelles, 2016), as is shown in the inset of Fig. 4. That plan can be described as a tube that became progressively subdivided over evolutionary time, into “neuromeres” along its rostro-caudal axis, and into radial sectors, including “alar” regions near the roof plate and “basal” regions near the floor plate. In caudal regions, the alar and basal regions correspond, respectively, to the dorsal and ventral horns of the spinal cord, but at the rostral end the tube has become highly expanded and curved such that the topology is challenging to recognize in modern brains. Nevertheless, the fundamental morphological unit is still a radial sector defined by gene expression gradients, where cell proliferation occurs at the ventricular surface prior to migration (Nieuwenhuys & Puelles, 2016).

This dramatic sequence of events culminated in the common ancestor of all chordates, a worm-like creature that lived during the Ediacaran epoch (635–540 Mya) and is believed to have resembled the modern amphioxus (Bertrand & Escriva, 2011; Lacalli, 2001, 2008; Wicht & Lacalli, 2005). Its nervous system was a tube with two main regions (Albuixech-Crespo et al., 2017). The rostral end was the “archencephalon,” most of which was derived from the legacy of the merged ANS/BNS. It still contained the chemo- and photosensitive cells and still controlled the global behavioral state through hormonal secretions, and most of it would eventually become what we now call the hypothalamus. The rest of the neural tube was the “deuterencephalon,” which derived from the BNS. It still controlled oscillatory contractions and would eventually become the hindbrain and spinal cord.

Extensive studies of the amphioxus (Holland & Holland, 1999; Lacalli, 2001, 2004, 2008; Putnam et al., 2008; Shimeld & Holland, 2005; Wicht & Lacalli, 2005) and larval tunicates (Lacalli & Holland, 1998; Ryan, Lu, & Meinertzhagen, 2016) suggest that the central nervous system of early chordates primarily consisted of a rudimentary hypothalamus attached to a locomotor hindbrain and a spinal cord that implemented undulatory swimming. In addition to controlling bodily physiology, the hypothalamic region controlled temperature- and light-dependent circadian activity patterns and simple control of filter-feeding behavior. The latter function involved shifting locomotor patterns from local exploitation to long-range exploration, on the basis of the richness of nutrient state signaled by dopamine (Hills, 2006), as described above.

**Fig. 5:**
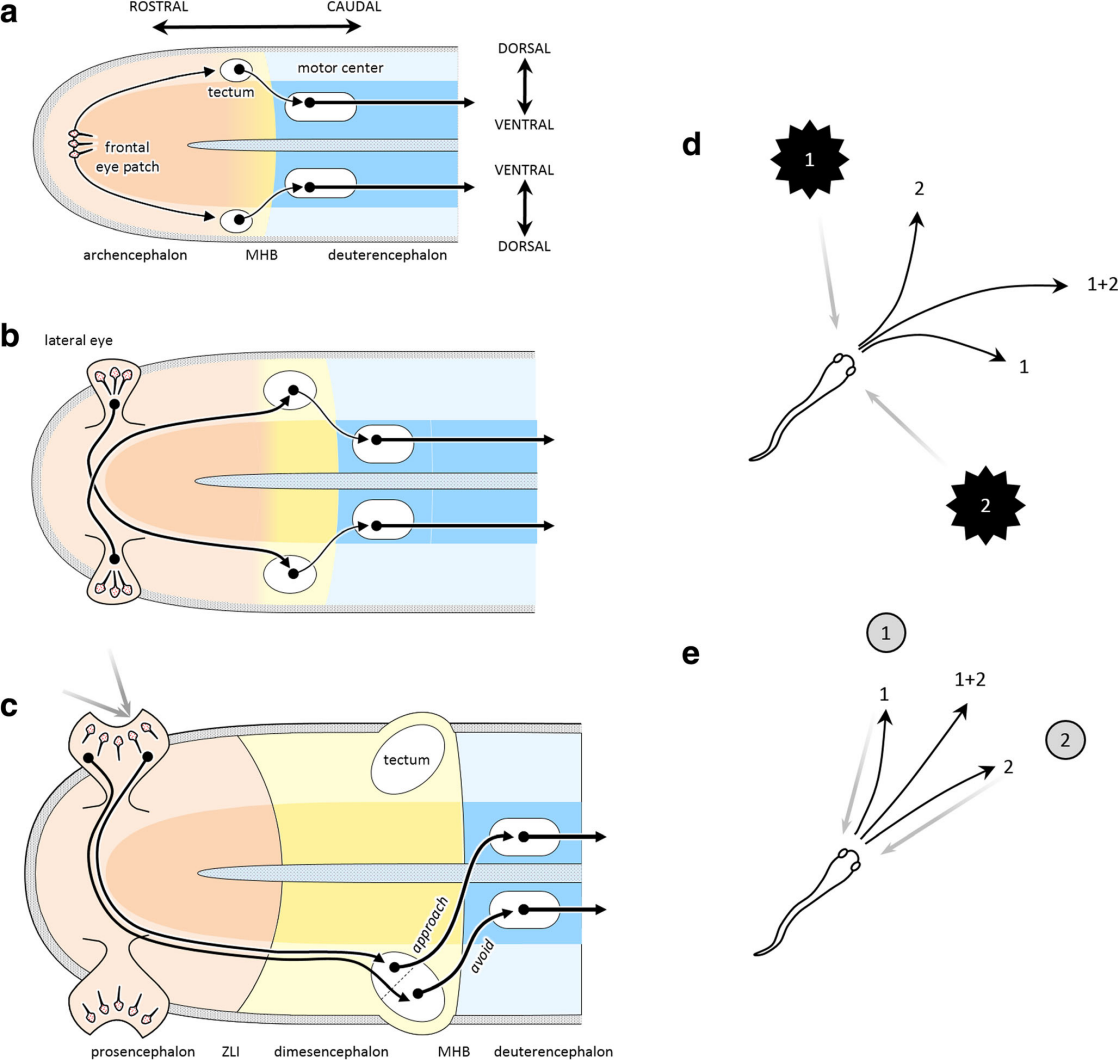
Evolution of avoidance and approach circuits. (A) Unfolded view of the neural tube of the putative last common ancestor of chordates. Escape behavior involved a single photosensitive patch of cells in the rostral tip, which projected bilaterally to the “tectum,” which projected ipsilaterally to basal “reticulospinal” neurons that controlled oscillatory locomotion. (B) In the cephalate, the eye patch split and moved to the lateral sides of the head, with contralateral projections to the tectum. (C) In early vertebrates, the eyes folded into cups, and the tectum differentiated to include a rostral region that projected contralaterally to the reticulospinal cells. This new circuit implemented visually guided orient-and-approach behavior. (D) In the presence of multiple threats (1 and 2), the averaging response (1 + 2) is effective in escaping from all of them. (E) Unlike escape, averaging between two stimuli for approach is maladaptive, making winner-take-all selection necessary. MHB, midbrain/hindbrain boundary; ZLI, zona limitans intrathalamica

An additional behavior, visually guided escape, was governed by a group of neurons in the alar portion of the caudal end of the archencephalon (Lacalli, 2018b), derived from the BNS, which would eventually become the midbrain (Fig. 5A). These received excitatory input from a central patch of photosensitive cells at the rostral tip of the neural tube and projected ipsilaterally to a basal set of neurons that stimulated fast undulatory swimming. Because the photosensitive cells fired in response to a reduction of light, whenever a shadow fell on the animal it would rapidly swim away. Gene expression data suggest that this circuit, still seen in the amphioxus, is homologous to the tectum of vertebrates, its retinal input, and its output projection to locomotor regions (Lacalli, 1996, 2006, 2018b; Shimeld & Holland, 2005; Vopalensky et al., 2012). Note that the escape circuit, when viewed from the perspective of a control system, still obeys the basic scheme of negative feedback control. Here, the impetus is caused by external conditions—a shadow that stimulates the photosensitive patch—and the response is escape, which moves the animal until the shadow is gone.

Gradually, a distinction formed within the archencephalon, between the rostral regions controlling nutrient balance and more caudal regions controlling escape behavior, leading to two distinguishable developmental domains: the ANS/BNS-derived “prosencephalon” (left ends of the images in Figs. 5A–5C) and the BNS-derived “dimesencephalon” (mid regions, in a different color) separated by the future site of the zona limitans intrathalamica (Albuixech-Crespo et al., 2017), a major gene expression boundary in brain development. This provides an example of how a single sensorimotor system gradually differentiated into two distinct segments that took on different functional roles: foraging versus escape. Nevertheless, due to their shared history, they retain similarities that provide explanatory power. In particular, they lay the foundations for what will later become the two main systems for visually guided behavior: a retino-tectal circuit for spatial orientation, and a retino-telencephalic circuit for advanced foraging and interaction.

About 540 Mya, the world’s fauna underwent a dramatic increase in diversity, called the “Cambrian explosion,” partially driven by advances in predation (Bengtson, 2002; Erwin et al., 2011). The ancestors of early vertebrates survived these tumultuous times through two advances in sensorimotor behavior. First, in what Ann Butler has called the “cephalate” (Butler, 2000), the single central photosensitive patch split into two patches that migrated to the sides of the head (Fig. 5B). The initially balanced visual input to the tectum continued to drive escape behavior, but over time projection patterns that were *contra*laterally biased proved most useful. That is because if a shadow fell on the left eye patch, it stimulated activity in the right tectum, which projected *ipsi*laterally to the locomotor region, causing the animal to first turn to the right before swimming away, akin to the “vehicles” of Braitenberg (1984). Conversely, leftward escape was initiated by a shadow falling on the right. As the eye patches expanded, they folded into cups and formed a lens (Lamb, 2013), resulting in a two-dimensional retina that provided a topographic mapping of external stimuli. The tectum expanded in parallel, with a matched topographic map of space in its superficial layers and gradients of downstream projections in its deep layers. The result was an “action map” of oriented escape responses to threatening stimuli at specific locations in the external world.

Microstimulation studies have revealed the presence of an organized map of oriented escape responses in the tectum of lamprey (Saitoh, Ménard, & Grillner, 2007), a jawless fish whose ancestors diverged from ours about 520 Mya. Different sites of stimulation induce rapid swimming, struggling, as well as contractions that produce downward shifts of the eyes and head and C-shaped body bending—in short, the types of species-specific behaviors that lamprey use to escape threats.

Microstimulation has also revealed a region in the rostral part of the lamprey tectum that produces behavior orienting *toward* objects—including eye and head turning followed by swimming. Interestingly, this region of the tectum receives input from a part of the retina that is sensitive to space in front of the animal with a complex collection of retinal ganglion cells, and it projects mostly *contra*laterally to the spinal cord (Jones, Grillner, & Robertson, 2009; Kardamakis, Saitoh, & Grillner, 2015). In short, it implements approach behavior (Fig. 5C).

We can describe the resulting architecture of the early vertebrate brain as a set of tectal circuits for different types of species-typical behaviors, each implemented as a closed feedback loop with the world aimed at eliminating the condition (“impetus”) that motivates it. A threat on our left motivates turning right so that the threat is behind us; a threat behind us motivates forward locomotion until it is gone. A food item in front motivates approach until the food is ingested and consumed. In each case, something about the world specifies an opportunity or a demand for action—what Gibson (1979) called an “affordance.” The neural activity in the circuit responding to that affordance is not a representation of a thing in the world; it is the specification of an action to take within the world. We can call it a “pragmatic representation” of action, as opposed to a “descriptive representation” of explicit knowledge (Cisek & Kalaska, 2010).

The presence of both approach and avoidance circuits raises the issue of behavioral selection: Given some stimulus in front of it, should the animal approach or escape? In lamprey (as in many vertebrates), that selection is performed within the tectum itself. For example, a small stimulus excites the cells involved in approach, which have a low threshold, but large looming stimuli excite the high-threshold cells that initiate escape (Kardamakis et al., 2015). More finely tuned selection could involve the detection of what ethologists call “key stimuli”—a set of specific cues that motivate a given action (Hinde, 1966). For example, frogs famously possess tectal “bug detectors” that combine converging information from specialized retinal cells sensitive to local sharp edges, dark spots with high curvature, fast motion signals, and local dimming (Lettvin, Maturana, McCulloch, & Pitts, 1959). In mammals, more sophisticated arbitration between approach and avoidance behavior is governed by descending modulation from the basal ganglia (Hormigo, Vega-Flores, & Castro-Alamancos, 2016).

In addition to the selection between approach and avoidance, a different type of selection is necessary within the approach system. Consider what happens when multiple stimuli are present simultaneously. If either or both of these are considered threats, then escape behavior is necessary, and in this case an average response is effective (Fig. 5D). However, that is not the case for approach, in which the average response would miss both targets. In this scenario, what is needed is a winner-take-all selection, whereby one response completely suppresses the other. This can be accomplished through lateral inhibition (Grossberg, 1973), a mechanism that can indeed be found in the lamprey tectum (Kardamakis et al., 2015).

To summarize, the lamprey tectum appears to contain the circuits for two kinds of spatially oriented behavior, which can broadly be described as “approach” and “avoidance.” Both receive contralateral input from the eyes, as in the ancestral cephalate. However, while the avoidance system retains the ancestral uncrossed output projections, in the approach system these projections are crossed. Importantly, these two systems appear to have been retained throughout vertebrate evolution. For example, in fish, stimulation of the optic tectum elicits eye movements and body bending, followed by several tail beats, whose orientation is either contraversive or ipsiversive, depending on the site of stimulation and current strength (Herrero, Rodriguez, Salas, & Torres, 1998). In rodents, stimulation of the superior colliculus (SC)—the mammalian homologue of the optic tectum—produces approach or avoidance actions through two distinct circuits (Comoli et al., 2012; Dean, Redgrave, Sahibzada, & Tsuji, 1986; Sahibzada, Dean, & Redgrave, 1986). Stimulation of the medial SC, which receives visual information from space above the animal and projects ipsilaterally to the brainstem and spinal cord, elicits defensive and avoidance actions. Stimulation of the lateral SC, which receives visual information from lower visual space as well as the vibrissae and projects contralaterally, elicits approach and appetitive behavior. This makes good sense in the world of rodents, in which predators often approach from above and food sources are found low to the ground. A similar distinction has also been found in the SC of primates. It is well known that the SC is implicated in the control of gaze and body orientation through contralateral projections to the brainstem (Basso & May, 2017), but its role in defensive behavior has only recently been demonstrated. In particular, chemical activation of the deep layers of the macaque SC evokes a dramatic increase in species-typical defensive behaviors, including cowering, escape, vocalization, and threatening gestures (DesJardin et al., 2013).

Alongside the elaboration of tectal visuomotor circuits of approach and avoidance, a second major advance in behavior during the early Cambrian epoch involved the elaboration of olfactory foraging systems. These originated in the rostral segment of the neural tube and involved the expansion of an olfactory region in the “alar” sector of the hypothalamus into what would ultimately become the telencephalon (Puelles, Harrison, Paxinos, & Watson, 2013). As we noted above in Fig. 3B, this ANS-related circuit was originally concerned with the control of nutrient concentration by arbitrating between local exploitation in nutrient-rich regions and long-range exploration away from nutrient-poor regions, governed by levels of dopamine (Hills, 2006), through its projections to “basal” locomotor centers. With advances in external chemical sensing, it was now possible to evaluate the nutrient environment before actual ingestion, and to use this to differentially bias actions. The early telencephalon was subdivided into two regions: the “pallium,” which implemented different olfactomotor actions (Derjean et al., 2010), and the “subpallium,” which arbitrated between them in the context of expected rewards (Redgrave, Prescott, & Gurney, 1999; Wickens & Arbuthnott, 2010). These regions would form the foundations from which the rest of the forebrain evolved. The subpallium became the striatum and pallidum of the basal ganglia, whose circuits are present in lamprey in all the detail so far studied (Grillner, Hellgren, Ménard, Saitoh, & Wikström, 2005; Robertson et al., 2014).

The differing demands of local exploitation versus long-range exploration led to the emergence of a distinction within the pallium between a ventrolateral sector (VLPall) that was specialized for exploitation and a medial sector (MPall) that was specialized for exploration. The ventrolateral sector used olfactory and gustatory signals, along with visual “key stimuli” arriving from the tectum via the “collothalamic” pathway (Butler, 2008), to guide appetitive approach actions, and would ultimately become the olfactory bulb, the insula, and piriform cortex. The medial sector, in contrast, used olfactory signals and direct “lemnothalamic” visual input (Butler, 2008) to guide navigation (Jacobs, 2012), and would eventually become the hippocampus (Jacobs & Schenk, 2003; Puelles et al., 2013).

Interestingly, part of the ventrolateral pallium of lamprey includes a retinotopic visual area and somatotopic areas receiving input from spinal and trigeminal systems (Suryanarayana, Pérez-Fernáandez, Wallén, Robertson, & Grillner, 2018), as well as a motor area from which microstimulation can evoke a rich repertoire of actions (Ocana et al., 2015). It is therefore topologically similar to the dorsal pallium (DPall) of jawed vertebrates, which is the homologue of the mammalian cerebral cortex (Butler & Hodos, 2005; Medina & Reiner, 2000). Although the similarity of these sensorimotor regions of the lamprey pallium to the mammalian cerebral cortex could be a product of convergent evolution, their detailed connectivity, synaptic properties, dendritic spine distribution, and neurotransmitters suggest they are a legacy of a circuit that existed in our last common ancestor (Ocana et al., 2015).

**Fig. 6:**
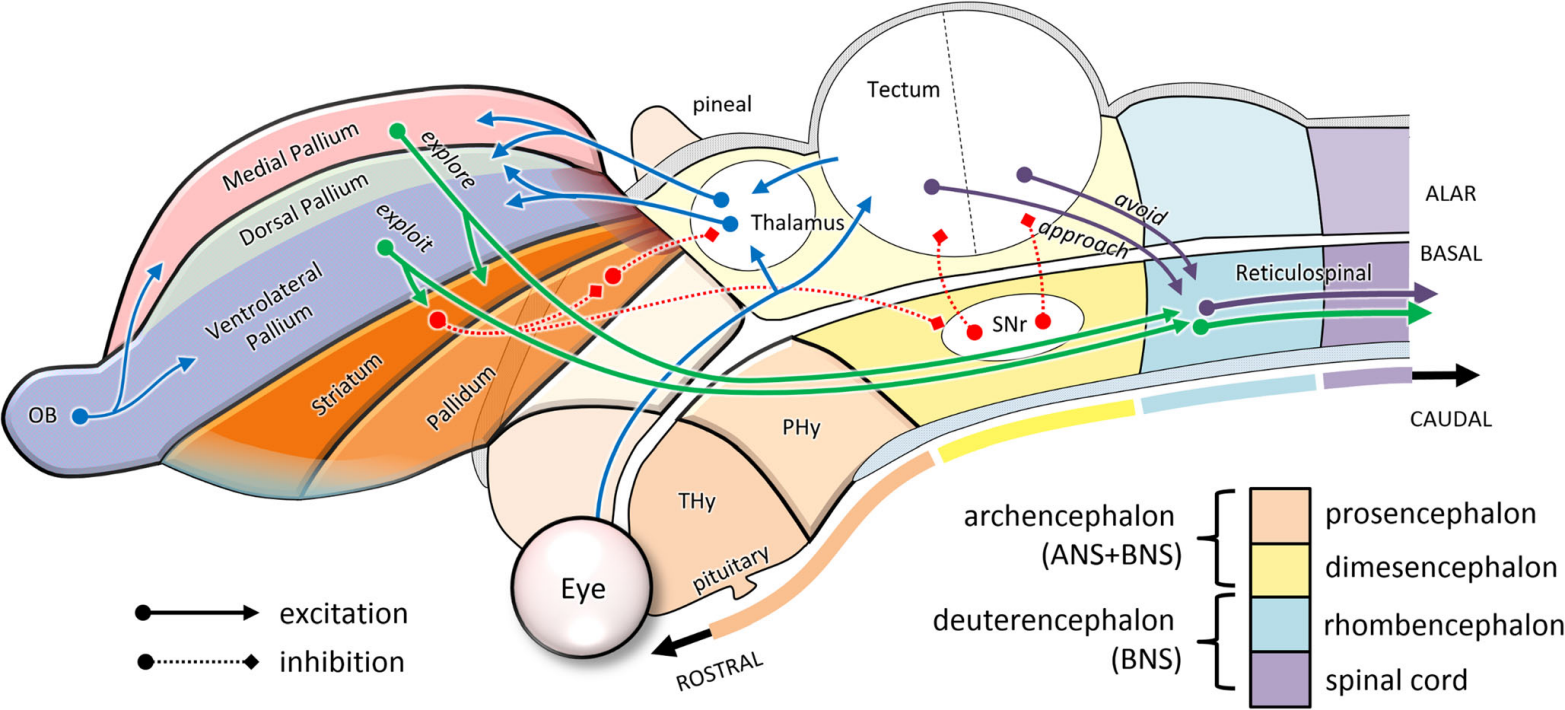
Sagittal view of the basic organization of the ancestral vertebrate brain. Here, the neural tube is color-coded according to its major subdivisions: prosencephalon, dimesencephalon, rhombencephalon, and spinal cord. The alar portion of the second segment of the prosencephalon (PHy) expands into the telencephalon, in which additional domains can now be distinguished. These include subpallial sectors (striatum and pallidum) and pallial sectors (ventrolateral and medial). The putative future site of the dorsal pallium is marked as a subregion of the ventrolateral pallium. Only a few of the major pathways are shown, emphasizing how visual and olfactory information (blue lines, in online color figure) is used to guide tectal approach and avoidance behaviors (purple lines), and telencephalic foraging behaviors (green lines), arbitrated by modulatory pathways from the subpallium (red dotted lines). OB, olfactory bulb; PHy, peduncular hypothalamus; SNr, substantia nigra reticulata; THy, terminal hypothalamus

To summarize, at the root of the vertebrate phylogenetic tree, the nervous system consisted of a tube divided into rostro-caudal neuromeres, as is shown in Fig. 6 on the basis of the prosomeric model of Luis Puelles and colleagues (Puelles et al., 2013; Puelles & Rubenstein, 2003). The most rostral neuromere was the top-level controller, influencing bodily physiology through secretions of hormones to the rest of the body, and behavior through neuromodulation of the rest of the neural tube. This became what Puelles et al. (2013; Puelles & Rubenstein, 2003) call the “terminal hypothalamus” (THy in the figure). The next segment, called the “peduncular hypothalamus” (PHy in the figure), was the top-level controller of simple foraging behaviors. It included what would become the lateral hypothalamic area and an expanded alar portion called the *telencephalon*. The latter part consisted of a ventrolateral pallial sector concerned with olfaction and ingestion (the future piriform cortex and insula), and a medial pallial sector concerned with navigation (the future hippocampus), orchestrated through an underlying subpallial system (basal ganglia) that arbitrated between different kinds of behaviors. These telencephalic systems implemented a set of parallel sensorimotor loops that received input through the thalamus (with the exception of direct olfactory input) and effected output through basal descending pathways to more caudal segments. The caudal segments included the midbrain, including the tectal approach and avoidance circuits, as well as the hindbrain and spinal cord, which implemented the control of undulatory locomotion.

The early vertebrate nervous system described above, which was present half a billion years ago, contained almost all of the basic pieces of mammalian brains, as well as their gross topological organization (Ocana et al., 2015; Robertson et al., 2014). One major innovation occurred between 500 and 450 Mya, with the elaboration of the alar hindbrain into what would become the cerebellum (Bell, Han, & Sawtell, 2008). Other major innovations occurred as vertebrates emerged onto land about 400 Mya (Lu et al., 2012), including the elaboration of the swim bladder into lungs, transformation of fins into legs, and development of the circuits controlling terrestrial locomotion. The complexity of life on land opened up many new opportunities and demands and encouraged a vast expansion of the behavioral repertoire. This included some elaboration of the retino-tectal circuits, but even more significant advances occurred through differentiation of the sensorimotor circuits lying at the border between the ventrolateral and medial pallia. This region became the dorsal pallium of amniotes, and eventually gave rise to what in mammals would become the cerebral cortex.

It is difficult to know why the dorsal pallium took on such a major role in the evolution of mammalian brains (Aboitiz, Morales, & Montiel, 2003). One reason might have to do with the nocturnal lifestyle of the early mammals, which reduced their dependence of vision and emphasized olfactory-driven foraging, thus motivating elaborations of the olfactory telencephalon. Another key factor may have been the nature of sensory projections from the thalamus, which enter the dorsal pallium of sauropsids (birds and reptiles) tangentially, whereas in mammals they enter radially, forming columns that can be duplicated and repeated without incurring major connectivity costs (Striedter, 2005). Thus, the dorsal pallium of mammals could grow dramatically into what we now call the *neocortex*, with each portion maintaining its ancestral connections with sensory input, descending output, and recurrent loops with the basal ganglia and cerebellum. The existing architecture of parallel sensorimotor streams, each specialized for one aspect of the animal’s behavioral repertoire, could be expanded and parcellated to support a wider range of behaviors. For example, in the context of foraging, local exploitation could expand from simple types of approach and ingestion behaviors to a great variety of sniffing, burrowing, reaching, and grasping behaviors.

**Fig. 7:**
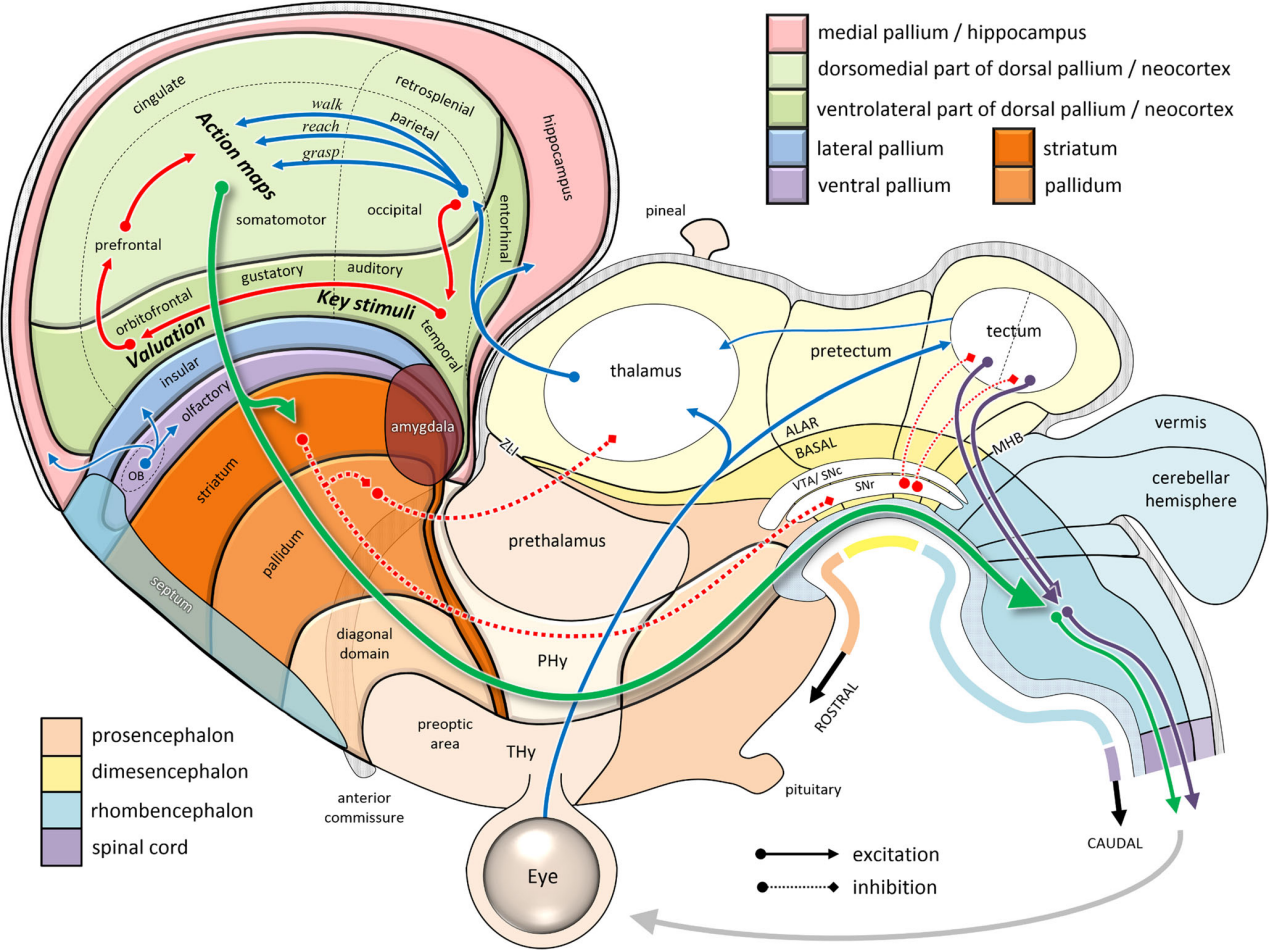
Schematic organization of the mammalian brain, based on Puelles et al. (2013). Here, the dorsal pallium (neocortex) has been divided into the spatially topographic (light) versus nontopographic (dark) neocortical sheets (Finlay & Uchiyama, 2015) and superimposed with labels based on the cortical flat map of Swanson (2000). Within the neocortical regions, blue arrows (see online color figure) indicate processes specifying potential actions, while red arrows indicate information related to their selection. Note the topological similarity of the tectal and telencephalic sensorimotor circuits to those shown in Fig. 6. OB, olfactory bulb; MHB, midbrain/hindbrain boundary; PHy, peduncular hypothalamus; SNc, substantia nigra compacta; SNr, substantia nigra reticulata; THy, terminal hypothalamus; VTA, ventral tegmental area; ZLI, zona limitans intrathalamica

Figure 7 shows an unfolded and flattened topological map of the mammalian brain, still respecting the major subdivisions of the ancient neural tube. Here we see how the neocortex lies within the dorsal pallial sector of the expanded alar subregion of the peduncular hypothalamus. Although it is unfamiliar to think of the cerebral cortex as a subregion of part of the hypothalamus, this topological placement reflects its role within the functional hierarchy of behavior: (1) The hypothalamus is the top-level controller of the general state of the organism. (2) Its second, “peduncular” segment PHy specializes in the kind of control that extends through the environment by means of downstream projections to the rest of the nervous system. (3) The alar portion of that segment specializes in the guidance of foraging, ranging from local exploitation via olfaction/ingestion (ventral and lateral pallium) to long-range exploration (medial pallium, a.k.a. hippocampus), with all the sensorimotor interactions in between (dorsal pallium). (4) With the expansion of opportunities for sensorimotor interaction afforded by the terrestrial world, this last part of the brain has expanded massively, especially in mammals, to form a neocortex consisting of multiple parallel sensorimotor streams.

In all mammals, the neocortex consists of two sheets (Finlay & Uchiyama, 2015), a dorsomedial sector that is spatially topographic and a ventrolateral sector that is nontopographic (see the green shaded areas in the online version of Fig. 7). In primates, the former includes dorsolateral prefrontal cortex, cingulate regions, all of premotor, motor, sensorimotor, and parietal cortex, as well as retrosplenial cortex. The latter includes orbitofrontal, gustatory, and visceral cortex, limbic cortex, and the temporal lobe. Much of the dorsomedial neocortex is organized into what Michael Graziano has called “action maps,” a set of fronto-parietal circuits dedicated to different classes of species-typical actions (Graziano, 2016; Kaas & Stepniewska, 2016). In early mammals (300 Mya), this was probably quite limited and consisted simply of medial circuits concerned with locomotion and lateral circuits concerned with head and mouth movements (Kaas, 2017). Each of these circuits processed sensory information in an idiosyncratic manner specialized for its specific type of action (e.g., space near the legs for locomotion, space near the snout for ingestion), and each projected to a specific set of relevant effectors. Meanwhile, the ventrolateral neocortex processed information relevant to selecting the type of action that would be most relevant at a given time (Cisek, 2007). This included interoceptive signals about the current physiological state, relayed via the insula, as well as simple mechanisms for detecting “key stimuli” (Hinde, 1966), similar to those already found in tectal circuits.

The ideas above are closely related to the “affordance competition hypothesis” (Cisek, 2007; Cisek & Kalaska, 2010; Pezzulo & Cisek, 2016), which suggests that the cortical control of behavior involves the parallel specification of different action opportunities currently available in the immediate environment and a competition between them that is biased by a variety of factors, such as object identity, expected rewards, and current behavioral context. That hypothesis, strongly inspired by the “two visual systems” view of Milner and Goodale (Goodale & Milner, 1992; Milner & Goodale, 1995), proposes that the specification of potential actions occurs in sensorimotor cortex (dorsal visual stream, dorsomedial cortical sheet) as a biased competition (Grossberg, 1973) within a recurrent network composed of groups of cells “voting” for different actions. The selection factors that influence that competition come from the basal ganglia and frontal regions using information from temporal cortex (ventral visual stream, ventrolateral cortical sheet). The present framework of the more fundamental organization of behavioral control systems provides a context within which that hypothesis naturally fits, but it also motivates some important modifications, as described below.

As the behavioral repertoire of mammals continued to expand, so did the dorsomedial neocortex, and there was a differentiation and specialization of action-specific maps of sensory space. In primates, the expansion of parietal cortex was particularly dramatic, yielding a variety of idiosyncratic representations of space particular to the needs of different action types (Andersen, Snyder, Bradley, & Xing, 1997; Stein, 1992). For example, visually guided reaching actions involve medial intraparietal cortex (Cui & Andersen, 2011; Kalaska, 1996), which represents targets within reach with respect to the direction of gaze and the position of the hand (Buneo, Jarvis, Batista, & Andersen, 2002; Gallivan, Cavina-Pratesi, & Culham, 2009) and is interconnected with the frontal regions controlling reaching, such as dorsal premotor cortex (Johnson, Ferraina, Bianchi, & Caminiti, 1996; Wise, Boussaoud, Johnson, & Caminiti, 1997). Grasp control involves the anterior parietal area (Baumann, Fluet, & Scherberger, 2009), which is sensitive to object shape and is interconnected with grasp-related frontal regions such as the ventral premotor cortex (Nakamura et al., 2001; Rizzolatti & Luppino, 2001). The control of gaze involves the lateral intraparietal area (Snyder, Batista, & Andersen, 2000), which represents space in a retinotopic frame (Colby & Duhamel, 1996; Snyder, Grieve, Brotchie, & Andersen, 1998) and is interconnected with frontal regions controlling gaze and with the superior colliculus (Paré & Wurtz, 2001).

The ventrolateral neocortex expanded also, particularly its caudal portion, where nonegocentric visual information was processed. In primates, this grew so much that the entire cortical hemisphere bent around the insula, eventually forming the familiar curled shape of the human brain. Those temporal regions, originally concerned with simple “key stimulus” detection, became elaborated into more sophisticated mechanisms sensitive to behaviorally relevant classes of objects in the world. Computationally, object recognition has been described as the “untangling” of low-level features into a high-dimensional space in which meaningful categories of external objects are more readily separable (DiCarlo & Cox, 2007). This need not end at the temporal lobe, however. What selection of behavior really needs is not representations of objects per se, but classification of the relative subjective value of engaging with those objects, as a function of the animal’s current state. That is, the ancestrally most relevant category is not “apple,” but “edible item,” perhaps contextually modulated by the current context of hunger, thirst, fatigue, and so forth. In other words, what behavior needs is cues that help prioritize one action over another. Yoo and Hayden (2018) proposed that the kind of untangling proposed to explain object recognition can be postulated to continue into orbitofrontal regions, which are often associated with representations of economic value (Padoa-Schioppa & Assad, 2006). This notion is compatible with the observation that orbitofrontal cortex indeed lies at the rostral end of that same ventrolateral neocortical sheet that starts with nonegocentric visual (and auditory) processing (Finlay & Uchiyama, 2015).

The resulting sketch of the organization of the cerebral cortex is consistent with the idea of affordance competition, but it suggests a more specific proposal on the division of labor between different *kinds* of selection problems (Cisek & Thura, 2018). One type of decision that an animal must make is what aspect of its behavioral repertoire should be engaged at a given moment. For example, if a desirable fruit is within reach, then one can engage the reaching system to grab it and bring it to the mouth, but if it is out of reach, then one must first engage locomotion. In each of these cases, the affordance is specified by visual information about the geometrical relationships between body effectors and the objects around them, the cues for selection are provided by visual information about shape and color, and the consequences of each candidate action are predictable due to the reliability of interactions with the environment, which in some cases (e.g., locomotion toward a fruit) will make a new affordance available (Pezzulo & Cisek, 2016). The first task for an animal is to selectively activate one of its fronto-parietal systems (Graziano, 2016), the one dedicated to the type of action that is called for (e.g., reach or walk). This type of “between-system” selection could be driven by the basal ganglia, given their anatomical placement as a central hub from the very origins of telencephalic sensorimotor control (Grillner & Robertson, 2015; Redgrave et al., 1999). However, once that type of selection is made, there is still a “within-system” competition that must be resolved—for example, between different fruits that could be grasped or different foot placements that are possible. This kind of selection is different: It requires a map of actions, in a continuous and idiosyncratic space specific to each type of action (hand-centered reachable space for reaching, retinotopic space for gaze, etc.)—that is, within each of the fronto-parietal action systems. For example, once reaching is chosen, specification of different targets for reaching could take place within a population of tuned cortical neurons in the fronto-parietal reaching system (MIP, PMd, M1), which implement a “desirability density function” across the space of reaching actions (Pezzulo & Cisek, 2016), and the competition could play out across that population simply through lateral interactions (Cisek, 2006; Grossberg, 1973). Several lines of evidence suggest that when choosing specific actions within a given class of actions, it is the cortex that makes the choice (Klaes, Westendorff, Chakrabarti, & Gail, 2011; Pastor-Bernier & Cisek, 2011; Thura & Cisek, 2014), not the basal ganglia (Arimura, Nakayama, Yamagata, Tanji, & Hoshi, 2013; Thura & Cisek, 2017; Turner & Desmurget, 2010).

The resulting functional architecture at which we have arrived is still fundamentally based on feedback control (Ashby, 1965; Cisek, 1999; Powers, 1973), whereby interaction with the world is aimed at exploiting available opportunities (“affordances”) that reliably reduce or eliminate some deviation from a desirable state (“impetus”). These feedback interactions exist on many hierarchical levels. Some are concrete actions, and because many are present simultaneously, selection must be made both between different types of actions (Cisek & Thura, 2018) and within specific movements of a given type (Cisek, 2007). These “low-level” control systems themselves make possible new domains of interaction, such that complex behavior can be constructed upon a scaffolding of simpler behaviors (Pezzulo & Cisek, 2016), extending even into social interactions (Hendriks-Jansen, 1996). For example, if you can predict how other animals will respond to your actions, you can extend your control through them. This can be used to explain a variety of social behaviors, from the threat postures of monkeys to a baby’s interactions with its mother. The concept of affordances can even be extended to a cultural domain (Ramstead, Veissiere, & Kirmayer, 2016), and within that context can provide much-needed grounding for theories of meaning in linguistic communication (Cisek, 1999). In short, it is possible, at least in principle, to extend the basic sketch of the functional architecture of simple sensorimotor control to the more abstract domains that characterize human behavior, as was proposed long ago by Piaget (1954). Exploring those possibilities, however, is beyond the scope of the present article.

## Conclusions

Readers who are primarily interested in human psychology may feel that this article ends just as things finally got interesting. All this time was spent describing the simple behavior of primitive animals, but almost no mention was made of such concepts as cognition, attention, working memory, conscious awareness, and so forth. There are two reasons for these omissions. First, the approach of phylogenetic refinement is necessarily chronological, so it constructs the story of brain elaboration in a stepwise fashion that must proceed from old to new, because each step provides the context for the next one. This article is just a first pass at sketching out such a story, and it will surely require frequent revision and correction. Not only do space limitations prevent me from going further, but so do the limitations of my own review of the literature, which has also been chronological. Nevertheless, although the result does not address many abilities of modern human brains, I hope that it may provide useful constraints for theories explaining those abilities, as will be discussed below.

Second, one of the primary purposes for following a chronological approach was to resynthesize the taxonomy of functional concepts that warrant explaining. If the concept of “cognition” has not entered the picture, this may be because we have not yet gone far enough, or it may be because cognition, as a dedicated and separate system, is a concept that is fundamentally incompatible with phylogenetic history. If the latter is true, then asking how “cognition” works may not be a biologically relevant question at all. At this point, the verdict is not yet clear. It may be that a serial cognitive “virtual machine” appeared in human brains atop their inherited architecture for flexible primate behavior (Block, 1995). However, I believe it unlikely that such a major redesigning of the functional architecture could have happened within the last few million years, after what had been nearly a billion years of relatively continuous differentiation and specialization of closed-loop feedback control systems. It seems more promising to consider how that architecture of nested feedback control, which has been extending further and further into the world for so long, might have just kept extending into increasingly abstract domains of interaction (Hendriks-Jansen, 1996; Pezzulo & Cisek, 2016). If so, this would lead to alternative explanations for many capacities often considered hallmarks of “cognition.”

**Fig. 8:**
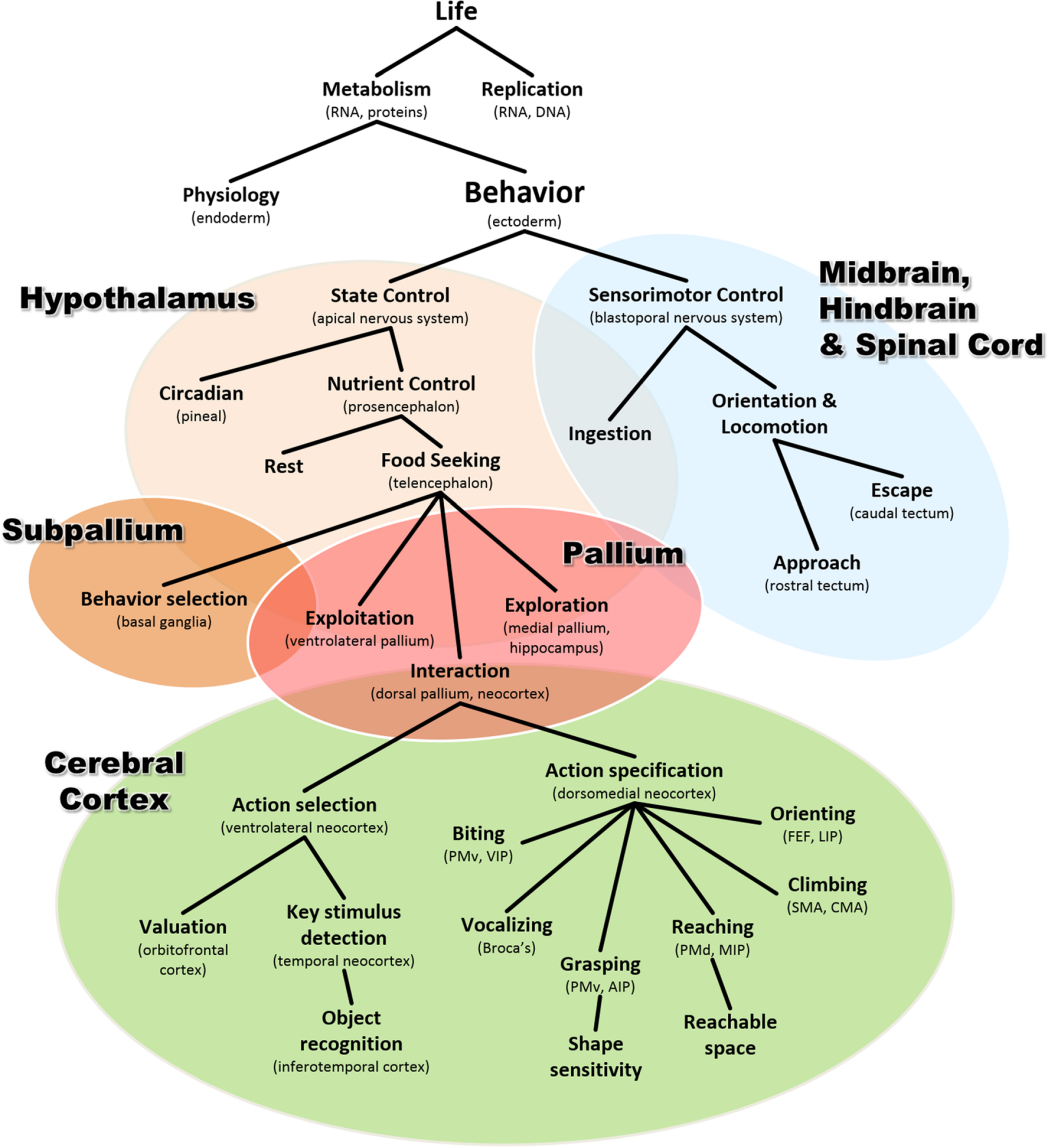
An alternative conceptual taxonomy resulting from following a phylogenetic approach along the vertebrate lineage. Here, each functional category is conceived as a particular specialization of the functional category above it, and each corresponds to a biological structure that emerged as a specialization within an ancestral structure. AIP, anterior intraparietal area; CMA, cingulate motor area; FEF, frontal eye fields; LIP, lateral intraparietal area; MIP, medial intraparietal area; PMd, dorsal premotor cortex; PMv, ventral premotor cortex; SMA, supplemental motor area; VIP, ventral intraparietal area

In summary, the chronological evolutionary story outlined above can be used to derive an alternative conceptual taxonomy, of which a draft example is illustrated in Fig. 8. Here, the hierarchy of functional categories is constructed by progressive differentiation that follows, along each branch, the putative sequence of specializations that occurred over evolutionary time. Thus, behavior is a specialization of metabolism, object recognition is a specialization of action selection, and so forth. I would argue that these are not simply semantic exercises, but useful constraints that can reveal similarities of the underlying mechanisms. For example, like all metabolic processes, behavior is organized as negative feedback control (Ashby, 1965; Cisek, 1999; Powers, 1973). Like other processes in the ventrolateral neocortex, object recognition and valuation share computational principles and are both ultimately used to bias the selection of actions (Cisek, 2007; Yoo & Hayden, 2018). One of the main advantages of this type of conceptual taxonomy is that it naturally maps into specific neural structures and captures the evolutionary and developmental relationships that determine how they specialized from each other and how they work together within modern brains.

Figure 8 is certainly very incomplete, and it is missing many of the familiar concepts of the classical taxonomy shown in Fig. 1. In some cases that is a symptom of a work in progress, but in others it may be indicative that the conceptual category itself does not correspond to any real biological entity. One example is “cognition,” as already discussed. Another example is “decision making,” which is usually seen as a cognitive process and categorized into economic, perceptual, emotional, or social decisions (Hastie, 2001; Heekeren, Marrett, & Ungerleider, 2008; Lee, 2008; Rangel, Camerer, & Montague, 2008). However, in the phylogenetic story outlined above, decision making appears many times and in very different contexts that have little to do with human cognition (Cisek & Thura, 2018). There are “hypothalamic” decisions between different behavioral states, such as sleep versus wake or feed versus rest. There are “tectal” decisions between approach and avoidance, and within the approach circuit, a spatial winner-take-all competition between targets. There are also “telencephalic” decisions between local exploitation and long-range exploration, “subpallial” decisions between different types of behaviors within one’s repertoire, and “neocortical” decisions between different movements within each type of behavior and between the different outcomes to which they may lead. In a search for the neural mechanisms of decision making, it seems important to define the problem in such a way as not to confound these distinct circuits or try to group them into arbitrary categories. In fact, there is no “decision-making system” in the brain, but instead a variety of selection mechanisms that gradually emerged within various circuits as behavioral sophistication gradually advanced.

Importantly, some of these selection mechanisms are responsible for phenomena often categorized as “attention,” such as the biased competition taking place within extrastriate visual areas (Boynton, 2005; Desimone & Duncan, 1995; Treue, 2001). In the case of the posterior parietal cortex, this has led to a persistent debate on whether this region is involved in representing attended objects (Bisley & Goldberg, 2010; Robinson, Goldberg, & Stanton, 1978) or intended action plans (Andersen & Cui, 2009; Andersen et al., 1997; Mountcastle, Lynch, Georgopoulos, Sakata, & Acuna, 1975). In the classical taxonomy (e.g., Fig. 1), “attention” and “intention” are distinct processes, one at the input to cognition and one at its output, so the posterior parietal cortex must be either one or the other, posing a dilemma regarding its functional role (Culham & Kanwisher, 2001). This dilemma does not apply to the phylogenetic perspective, in which the selection of spatial information in the posterior parietal cortex is simply a necessary arbitration between the many things a sophisticated animal can do in its complex niche. It is related to the selection challenge faced by the approach circuit of the tectum (e.g., Fig. 5E), another region often interpreted as a node in an “attentional network.” But “attention,” as a biological category, does not exist (Anderson, 2011; Hommel et al., 2019; Hommel & Colzato, 2015).

Another persistent debate—the question of representations—can also be viewed through the lens of a phylogenetic approach. Clearly, internal states that correspond to the external world are useful for even the simplest control systems. The neural activity at a particular site on a tectal map must specify the location of an object in space if it is to be used to guide approach toward that object. However, it need not be an explicit “descriptive representation” that, when properly decoded, yields information about the identity of that object (Brette, 2019). It merely needs to help determine whether the object is worth approaching, and if so, to specify the initial state of a dynamical system that flows toward the state in which the object is ingested. We can think of it as a “pragmatic representation” whose role is not to describe the world but to guide interactions with the world. Similarly, the neural activity in the parietal cortex of a monkey need not be an explicit description of space, but instead a functionally motivated mixture of information about potential targets for action, modulated by the behavioral relevance (Mountcastle, Andersen, & Motter, 1981) and subjective desirability (Dorris & Glimcher, 2004) of those actions. Even in the ventral visual stream, neural activity sensitive to visual features may not be a purely objective encoding of objects in the world, but may simply detect key stimulus cues for selection (Tanaka, Saito, Fukada, & Moriya, 1991), modulated by their behavioral relevance (Boynton, 2005; Treue, 2001). It thus may play a role not as a representation of the world but as the front end of mechanisms for deciding between different ways of acting in the world (Cisek, 2007; Yoo & Hayden, 2018).

Nevertheless, within the context of an evolutionary perspective, it is possible to conceive of how more explicit descriptive representations could gradually have emerged from ancestral pragmatic ones. In particular, immediate behavior emphasizes neural activities that combine information about the external world with information about current internal needs, to construct an impetus that motivates specific actions. However, more abstract and high-level behavior might benefit by differentiating and specializing these activity patterns, such that some of them become divorced from information about the current internal state and can be used to guide future behavior when the internal state changes. For example, the medial pallium of early vertebrates implemented long-range exploration driven by the impetus of being in a low-nutrient environment and developed mechanisms for learning how to navigate to a better environment (Broglio et al., 2005; Ocana, Uceda, Arias, Salas, & Rodriguez, 2017; Rodriguez et al., 2002). These mechanisms involved learning the relationships between the animal’s movement and changes in relevant sensory inputs, such as odor gradients and visual landmarks (Jacobs & Schenk, 2003). Presumably, these learning mechanisms initially operated exclusively in the pragmatic service of reducing hunger, but over time they could have become divorced from their motivational context and continued to construct cues for navigation, even while the animal was not hungry and not foraging. Thus, the animal would begin to build a kind of knowledge of its surroundings, akin to what could be called a “cognitive map” (Jacobs, 2012; Tolman, 1948), which could later be used to find food. This would be an example of a descriptive representation, albeit within the context of navigation and not “cognition,” arising in what ultimately became the hippocampus. Importantly, if we consider how descriptive representations of the world emerged within a given pragmatic context, then we will always keep them connected to their functional relevance—their *meaning*. Thus, a phylogenetic approach can even be applied to purely abstract representations, with the benefit of never suffering from the symbol-grounding problem (Cisek, 1999; Harnad, 1990).

An evolutionary perspective can also be a powerful “litmus test” for candidate theories about behavior. Many explanations have been proposed for how neural mechanisms implement complex behavior, but most of these are aimed at the abilities of modern primates, such as object recognition (Riesenhuber & Poggio, 2002), cognitive control (E. K. Miller & Cohen, 2001), or memory (Squire, Wixted, & Clark, 2007), and rarely do they address the question of how the proposed mechanisms could have been constructed by evolution. In some cases, the prospects for evolutionary plausibility seem unlikely. For example, it has been proposed that the human motor system is continuously optimizing, at each moment during movement, a cost function across all possible future paths to the goal state (Todorov, 2004). This is an extraordinarily computationally demanding task, and while the modern primate brain may have the power to implement it, that seems unlikely to be the case for the humble amphioxus. This then raises the question of what plausible sequence of continuous elaboration could have led from the simple feedback control in prevertebrates to cost optimization mechanisms in primates. At what phylogenetic stage would the system develop a representation of a cost function and reorganize its control mechanisms around minimizing that function? I don’t claim that a phylogenetic sequence *could not* be proposed for how this mechanism evolved, but to my knowledge this has not yet been done, and such a proposal would be necessary before the theory can be considered biologically plausible.

Other models are more compatible with phylogenetic constraints, and this is particularly true of those already constrained by a wide range of neuroanatomical, neurophysiological, and behavioral data. For example, Steve Grossberg and colleagues have developed a wide range of models of how cortical and subcortical circuits implement motivated behavior (Dranias, Grossberg, & Bullock, 2008), visual object recognition (Fazl, Grossberg, & Mingolla, 2009; Grossberg, Kuhlmann, & Mingolla, 2007; Grossberg, Srinivasan, & Yazdanbakhsh, 2011), learning and memory (Grossberg, 2018), navigation (Browning, Grossberg, & Mingolla, 2009), and many other phenomena. In some cases, the models are progressively constructed in a stepwise manner that is meant to reflect a plausible evolutionary sequence of how natural selection might have modified the nervous system toward increasing functional sophistication (Bullock & Grossberg, 1990; Grossberg, 1978). For example, Dranias, Grossberg, and Bullock 2008 described a model of motivated behavior and learning that consists of components corresponding to the lateral hypothalamic area, basolateral amygdala, ventral striatum and pallidum, pedunculopontine nucleus, ventral tegmental area, striatum, and medial and lateral orbitofrontal, rhinal, and inferotemporal cortex. All of these are connected in a realistic network whose elements exhibit activity patterns that simulate a wide range of neurophysiological results. Although the authors focused primarily on the full modern circuit, they also proposed a stepwise elaboration in which individual elements were introduced so as to gradually expand functional capacity (see their Fig. 7). This was not explicitly guided by data on the phylogenetic history of the relevant structures, yet the proposed sequence is at least potentially compatible with those data. It begins with a primarily “hypothalamic” circuit for matching drives and unconditioned stimuli, extends it with a “pallial” mechanism for learning conditioned stimulus associations, expands an appetitive-versus-aversive subcircuit corresponding to basolateral amygdala and subpallium, and finally adds components corresponding to orbitofrontal and inferotemporal cortical regions. Thus, although many phylogenetic details are not considered in their exposition, the Dranias et al. model is a good example of the kind of theory at which one might hope to arrive, after having followed an approach of refinement strictly constrained by phylogenetic data.

The present article has focused almost exclusively on sensorimotor control, in part under the assumption that the fundamental role of the nervous system is to endow animals with a means of adaptively interacting with their world. My hope is that I have demonstrated how a method of gradual phylogenetic refinement, constrained by comparative and developmental data, can be used to synthesize a theoretical framework of neural organization (e.g., Fig. 7) that potentially reflects the taxonomy of true functional categories (e.g., Fig. 8) better than do the classical concepts of perception, cognition, and action. In vertebrates, this includes tectal circuits for oriented approach and avoidance, as well as telencephalic systems for controlling exploitation, exploration, and a range of species-typical interactions. Admittedly, I have entirely neglected many important phenomena, some of which could themselves be phylogenetically fundamental. In many cases this is simply a limitation of scope, and does not imply that such phenomena cannot be addressed through an evolutionary method. In fact, several books have done just that. For example, Murray et al. (2017) used an evolutionary approach to resynthesize concepts of memory. Passingham and Wise (2012) have reconstructed prefrontal function in the context of the foraging strategies of primates. And Feinberg and Mallatt (2016) even used such an analysis to address the question of consciousness.

In conclusion, many psychological phenomena can be entirely deconstructed from a phylogenetic perspective and resynthesized in a rather different form. Some of these might challenge widely held assumptions about the elements of behavior, such as distinctions between perception, cognition, and action (Firestone & Scholl, 2016; Fodor, 1983; Pylyshyn, 1984) and between concepts such as “working memory” and “attention.” But this does not imply that we throw away data from experiments inspired by those concepts; indeed, evolution might provide a better context for interpreting the data, especially at the level of neural circuits. We all recognize that the long and tumultuous history of philosophical and psychological thought has led cognitive science and neuroscience to a variety of assumptions and stances, and that it is likely that some of these are as incorrect and misleading as other products of history that we have already rejected (e.g., dualism). This article argues that part of a strategy for moving forward is to seek insights from another kind of history, that of the evolution of our species.

### Author note

The author is supported by the Canadian Institutes of Health Research (MOP-102662), the Natural Sciences and Engineering Research Council of Canada (RGPIN/05245), and the Fonds de la recherche en santé du Québec. This article is part of a special issue inspired by the 2018 Leading Edge Workshop of the Psychonomic Society, organized by Joo-Hyun Song and Timothy Welsh, and I thank the organizers for the honor of being invited to take part. This work is a theoretical review and does not have any associated experimental data.
